# Comparison of spectral domain and swept source optical coherence tomography for angle assessment of Chinese elderly subjects

**DOI:** 10.1186/s12886-019-1145-7

**Published:** 2019-07-08

**Authors:** Yunsheng Qiao, Chen Tan, Min Zhang, Xinghuai Sun, Junyi Chen

**Affiliations:** 1 Department of Ophthalmology & Visual Science, Eye and ENT Hospital of Fudan University, 83 Fenyang Rd, Shanghai, 200031 China; 2 State Key Laboratory of Medical Neurobiology, Institutes of Brain Science and Collaborative Innovation Center for Brain Science, Fudan University, Shanghai, 200032 China; 3 NHC Key Laboratory of Myopia (Fudan University), Key Laboratory of Myopia, Chinese Academy of Medical Sciences and Key Laboratory of Visual Impairment and Restoration of Shanghai, Shanghai, 200031 China

**Keywords:** Optical coherence tomography, Spectral domain, Swept source, Angle, Wavelength

## Abstract

**Background:**

This comparative study aimed to demonstrate the differences between swept source OCT (SS-OCT) (1310 nm) and spectral domain OCT (SD-OCT) (840 nm) for the identification and measurement of anterior chamber angle (ACA) structures.

**Methods:**

Sixty seven eyes from 67 healthy subjects underwent ACA imaging at the nasal and temporal sides using SS-OCT and SD-OCT with different wavelength (Tomey, 1310 nm and RTvue, 840 nm). Images were evaluated for the ability to distinguish angle structures including the Schwalbe’s line (SL), the Schlemm’s canal (SC) and the scleral spur (SS). The length of trabecular meshwork (LTM), the angle-opening distance (AOD500 and AOD750) and the length of Schlemm’s canal (LSC) were also measured.

**Results:**

The nasal identification rate for SL, SC and SS were 91.04%/89.55%, 50.75%/40.30% and 100.0%/74.63% (SS-OCT/SD-OCT), respectively. The temporal identification rate for SL, SC and SS were 86.57%/91.04%, 68.66%/70.15% and 100.0%/65.67% (SS-OCT/SD-OCT), respectively. Differences between SS-OCT and SD-OCT were found in terms of the visualization of the SS. With respect to the measurements of angle, the evaluation of LTM at the nasal side, LSC at the temporal side and AOD500/750 at both sides showed significant difference between the two devices. However, there existed good correlation between the AOD500/750 measured by SS-OCT and SD-OCT (Spearman’s rank correlation coefficient > 0.8, *p* < 0.000).

**Conclusions:**

SS-OCT displayed a better performance in detecting deeper structures of the angle such as the SS. However, for discriminating structures lying in transparent or semi-transparent tissue such as the SL and the SC, the two devices showed good consistency. Although SS-OCT and SD-OCT demonstrated high correlation for angle measurement (AOD500/750), their agreement was poor.

## Background

The assessment of the ACA is essential for diagnosis and treatment of glaucoma. Remaining the gold standard for the evaluation of ACA, the gonioscopy permits direct visualization of angle structures through microscope-aided eyes. However, the evaluation is relatively subjective and falls short of precision.

The introduction of ultrasound biomicroscopy (UBM) and anterior segment optical coherence tomography (AS-OCT) paved the way for precise angle measurement. Compared to UBM, AS-OCT provides noncontact, in vivo imaging of ACA together with other benefits such as higher axial resolution and shorter acquisition time [1, 2].

The application of Fourier-domain OCT (FD-OCT) was expanded in the field of angle assessment with higher resolution and scanning speed in contrast to time-domain OCT (TD-OCT). FD-OCT can be further divided into spectral-domain OCT (SD-OCT) and swept-source OCT (SS-OCT). SD-OCT devices use a broadband near-infrared superluminescent diode as the light source with a spectrometer as the detector. In this discussion, we chose RTVue (Optovue Corporation, Fremont, CA) with a central wavelength of around 840 nm [3]. On the other hand, SS-OCT instruments apply a tunable swept laser as the light source with a single photodiode detector. SS-1000 CASIA (Tomey, Nagoya, Japan) has been selected as the representative with the wavelength centered approximately 1310 nm [3]. The difference of operating mechanisms inevitably leads to the disparities of imaging quality and detecting capability between the two subtypes of FD-OCT which requires further demonstration and clarification. The purpose was to compare SD-OCT and SS-OCT concerning the identification and measurement of angle structures as well as evaluate the correlation and agreement between the two.

## Methods

In this cross-sectional study, sixty-seven healthy Chinese elderly subjects were recruited for ACA evaluation from 11, November to 23, December, 2016 at the Eye and ENT Hospital of Fudan University, Shanghai, China. All subjects underwent a series of ocular evaluations including a detailed medical history taking, slit-lamp biomicroscopy, refraction examination, A-scan, ultrasound biomicroscopy, Goldmann applanation tonometry, gonioscopy, dilated fundus examination and standard automated perimetry. The inclusion criteria are: normal-appearing anterior segment, open ACA, intraocular pressure between 10 and 21 mmHg, normal fundus appearance and no sign of visual field defect. Subjects with best-corrected visual acuity of ≤20/40, spherical refractive error > + 3 or < −3D, axial length > 25 mm or < 19 mm, evidence of peripheral anterior synechiae on indentation by gonioscopy, previous use of any topical or systemic medication that could affect the aqueous humor circulation, history of intraocular surgery or penetrating trauma, laser trabeculoplasty, laser iridotomy, or laser iridoplasty were excluded from the study. When both eyes of the same subject were qualified, one eye was selected randomly.

### Anterior segment OCT imaging

**Fig. 1:**
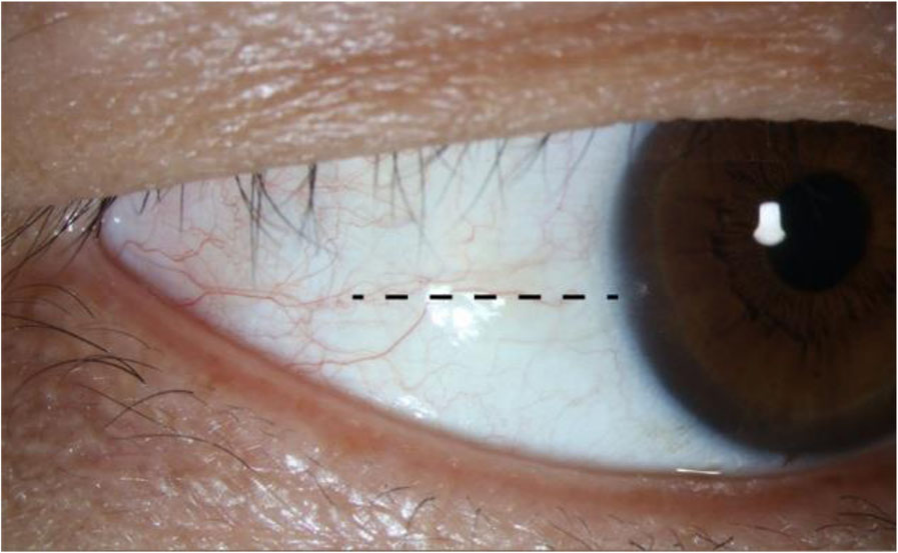
A picture demonstrates the temporal corneal limbus. The black dotted line indicates the conjunctival vessel chosen as the anatomical landmark for OCT examination

**Fig. 2:**
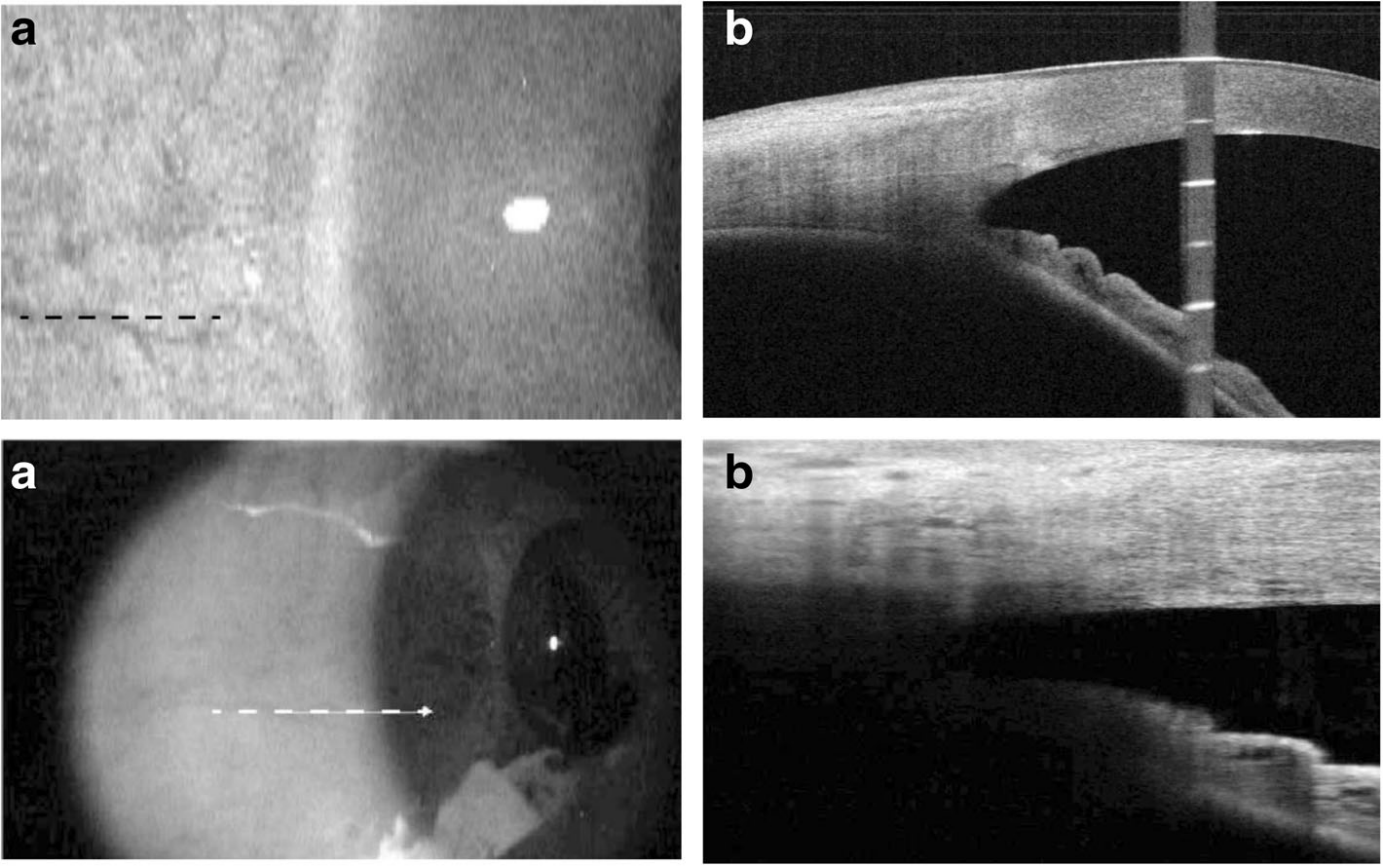
OCT imaging of the same location of anterior chamber. When using SS-OCT, we firstly identified the vessel chosen in Fig. 1 as indicated by black dotted line (**a**), and then took the cross-sectional image (**b**). Similarly, the same vessel was located under SD-OCT as was outlined by white dotted line in (**c**) to make sure that the image (**d**) shows the same part of the anterior chamber

Anterior segment imaging was performed under dark conditions by a single trained examiner (JYC) masked to clinical findings. The two OCT devices were set in the same dark room. The order of examination was randomly decided. For each subject, a certain anatomical mark (e.g. a conjunctival vessel or pigmentation) was chosen to make sure that the same part of ACA was assessed and compared (Fig. 1 and Fig. 2). The external target light of each instruments was used to direct the patients’ fixation. The brightness of the two fixation lights was measured by commercially available photometer [UT383 (UNI-T, Guangdong, China)] and no significance of statistical difference was found (Mean brightness for SS-OCT and SD-OCT were 75.0 and 77.4, *p* value = 0.334). Figures of the nasal and temporal angles (3 and 9 o’clock positions) were obtained according to the anatomical mark. At least three images were obtained for a single quadrant, the one with the clearest visibility of angle structures was chosen for further evaluation. As for SD-OCT, a CAM-L lens (cornea lens adapter; Optovue, Inc.) was mounted over the imaging aperture. The SD-OCT imaging was performed according to the CL Angle protocol (software version 4.0.7.5; RTVue OCT; Optovue, Inc., Fremont, CA). As for SS-OCT, the Angle (HD) (software version 6A; SS-1000 CASIA, Tomey, Nagoya, Japan) protocol was used to capture images.

### Image analysis

The following angle structures were of interest and identified:

1. Visualization of the SS: The SS in anterior segment imaging is marked by a prominent inner extension of the sclera and represents an anatomical landmark for the junction between the inner wall of the trabecular meshwork and the sclera [4].
2. Visualization of the SL: The SL is defined as the posterior limbal zone bordering the cornea where Descemet’s membrane terminates [5].
3. Visualization of the SC: The SC is seen as a curvilinear lucent area external to the trabecular meshwork. This lucent area extended from the scleral spur to the anterior tip of the trabecular meshwork located at the end of the Descemet’s membrane [6].
4. The LTM was defined as the distance from the Schwalbe’s line to the scleral spur.
5. AOD500 is the distance between a point of the cornea which is 500 μm away from the scleral spur and the opposite point of the iris [7].
6. AOD750, likewise, is the distance between a point of the cornea which is 750 μm away from the scleral spur and the opposite point of the iris.
7. The LSC was defined as the distance from the highest to the lowest point measured from the cross-sectional image of the Schlemm’s canal.

**Fig. 3:**
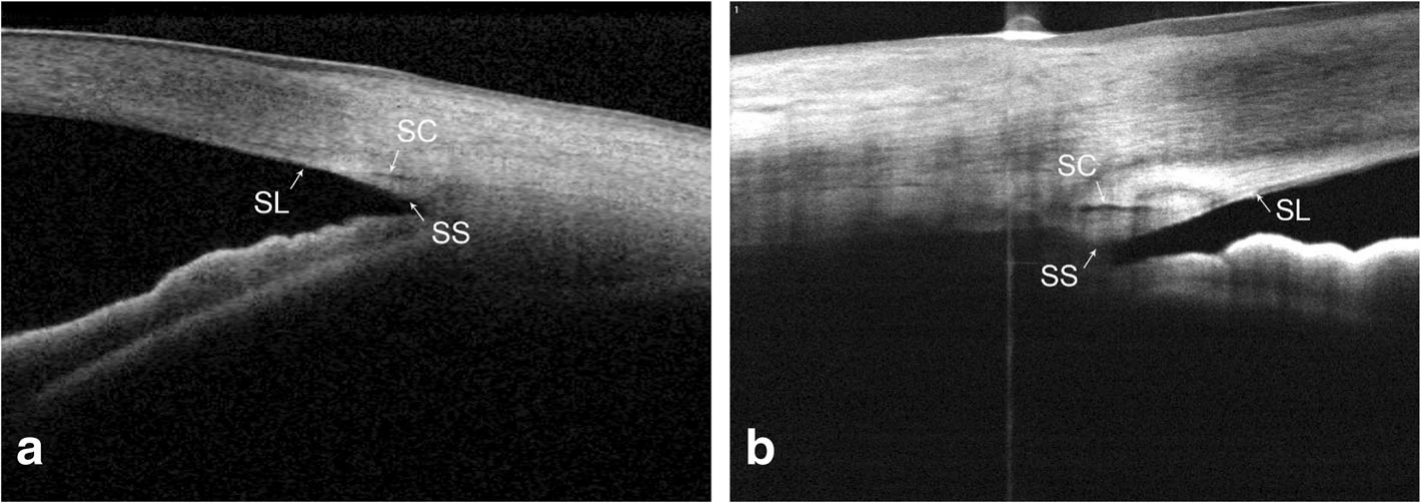
Cross-sectional images of SS-OCT (**a**) and SD-OCT (**b**) Pictures of iridocorneal angle were obtained during OCT examination, and angle structures are demonstrated. (SC = Schlemm’s canal; SS = scleral spur; SL = Schwalbe’s line)

**Fig. 4:**
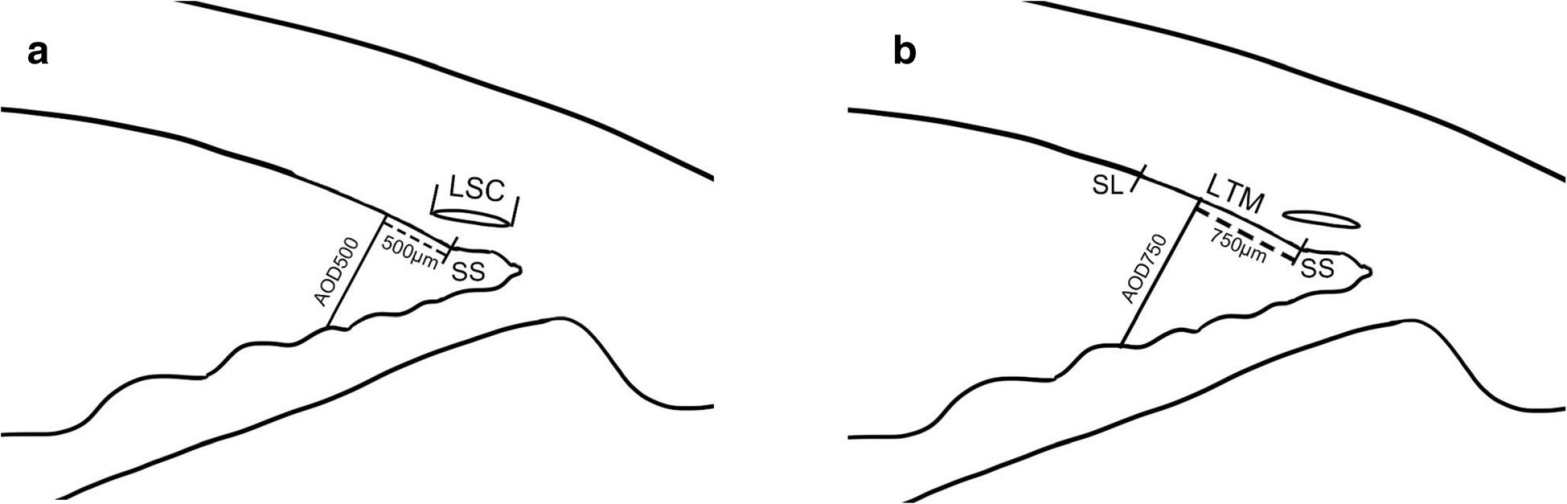
The illustrations of angle measurement (LSC = the length of the Schlemm’s canal; LTM = the length of the trabecular meshwork; AOD = angle opening distance; SS = scleral spur; SL = Schwalbe’s line)

The nasal and temporal angles were both measured in this study. An illustration of identification and measurement of structures is depicted in Fig. 3 and Fig. 4. All OCT scans were analyzed separately by two examiners (JYC, YSQ) masked to clinical findings, and the interobserver reproducibility for the angle assessment was evaluated in a random selection of 30 images. The calculated intraclass correlation coefficient (95% CI) was 0.98 (0.97–0.99).

### Statistical analysis

For the continuous variables, data obtained from two OCT scans were first examined by Kolmogorov-Smirnov test for the normality of distribution. For those variables which complied with normal distribution, paired Student’s t test was used to compare the difference between measurements made by two OCTs and the disparity between nasal and temporal data measured by the same device. Otherwise, Wilcoxon rank-sum test was applied to do the comparison. The correlation and agreement between SS-OCT and SD-OCT were evaluated by Pearson correlation coefficients (or Spearman’s rank correlation coefficient, based on the normality of distribution) and Bland-Altman plots, respectively. As for the dichotomous variables, Fisher’s exact test was adopted to analysis the measurements of two OCT scans. Statistical analysis were performed with SPSS software (version 24.0, IBM Corp.). A *P* value less than 0.05 was considered statistically significant.

## Results

The study comprised 67 eyes of 67 healthy subjects among which 37.3% (25) were male and 62.7% (42) were female. The mean ± SD age was 60.98 ± 7.76 years.

### Identification of angle structures

#### SS-OCT

Using SS-OCT OCT, the SS, SL, and SC were identified in 67(100%)/67(100%), 61(91.04%)/58(86.57%), and 34(50.75%)/46(68.66%) of subjects at the nasal and temporal quadrants, respectively. (Table 1).

**Table 1:** Comparison of angle structures discerning ability between SS-OCT and SD-OCT

Structure		TOMEY		OPTOVUE		*P* value
		*N* = 67	%	N = 67	%	
SS	(N)	67	100.00	50	74.63	< 0.0000
	(T)	67	100.00	44	65.67	< 0.0000
SL	(N)	61	91.04	60	89.55	1.0000
	(T)	58	86.57	61	91.04	0.6072
SC	(N)	34	50.75	27	40.30	0.2810
	(T)	46	68.66	47	70.15	1.0000

*SS* Scleral spur, *SL* Schwalbe’s line, *SC* Schlemm’s canal

#### SD-OCT

Using SD-OCT OCT, the SS, SL, and SC were identified in 50(74.63%)/44(65.67%), 60(89.55%)/61(91.04%), and 27(40.30%)/47(70.15%) of subjects at the nasal and temporal quadrants, respectively. (Table 1).

Visualization of the SS from both nasal and temporal quadrants was achieved by SS-OCT. Both devices presented satisfying detection rate for the SL. And the difference between the two OCTs for the same quadrant was not statistically significant (*p* = 1.0000 for nasal side, *p* = 0.6072 for temporal side), either as the two quadrants measured by the same device (*p* = 0.5488 for SS-OCT, p = 1.0000 for SD-OCT). Whereas, the identification of the SC was not that fulfilling, and both OCTs made better performance in identifying the SC from temporal side than that from nasal side (*p* = 0.0290 for SS-OCT, *p* = 0.0022 for SD-OCT) .

### Measurement of angle structures

#### SS-OCT

Using SS-OCT, the LTM, AOD500, AOD750 and the LSC were 702.49 ± 108.32 μm /669.84 ± 100.21 μm, 283.64 ± 128.56 μm / 333.99 ± 150.12 μm, 391.33 ± 176.86 μm / 462.30 ± 216.61 μm, and 225.91 ± 41.44 μm / 243.85 ± 43.34 μm at the nasal and temporal quadrants, respectively. (Table 2).

**Table 2:** Comparison of angle structures measurements between SS-OCT and SD-OCT

		TOMEY	OPTOVUE	P value	Correlation	*P* value^c^
LTM	(N)	702.49 ± 108.32	657.08 ± 112.15	0.002	0.510	0.000
(μm)	(T)	669.84 ± 100.21	648.67 ± 101.82	0.137	0.418	0.009
AOD500	(N)	283.64 ± 128.56	314.98 ± 148.52	0.000^a^	0.897^b^	0.000
(μm)	(T)	333.99 ± 150.12	370.36 ± 186.85	0.000^a^	0.811^b^	0.000
AOD750	(N)	391.33 ± 176.86	424.06 ± 196.32	0.000^a^	0.927^b^	0.000
(μm)	(T)	462.30 ± 216.61	491.93 ± 255.04	0.009^a^	0.811^b^	0.000
LSC	(N)	225.91 ± 41.44	215.70 ± 50.82	0.074	0.704	0.003
(μm)	(T)	243.85 ± 43.34	215.83 ± 37.99	0.007	0.093	0.597

*LTM* Length of trabecular meshwork, *AOD500* Angle opening distance 500 μm, *AOD750* Angle opening distance 750 μm, *LSC* Length of Schlemm’s canal

^a^ Wilcoxon rank-sum test

^b^ Spearman’s rank correlation coefficient

^c^ This P value is for correlation

#### SD-OCT

Using SD-OCT OCT, the LTM, AOD500, AOD750 and the LSC were 657.08 ± 112.15 μm /648.67 ± 101.82 μm, 314.98 ± 148.52 μm / 370.36 ± 186.85 μm, 424.06 ± 196.32 μm / 491.93 ± 255.04 μm, and 215.70 ± 50.82 μm / 215.83 ± 37.99 μm at the nasal and temporal quadrants, respectively. (Table 2).

Notably, there were significant difference in AOD500 (*p* < 0.000 for nasal and temporal side) and AOD750 (p < 0.000 for nasal and *p* = 0.009 for temporal side) between SS-OCT and SD-OCT. Significant difference of AOD500 and AOD750 also existed between nasal and temporal sides regardless of the measuring instrument (all p < 0.000). Additionally, the LTM and LSC in the nasal side was longer when evaluated by SS-OCT (*p* = 0.002, *p* = 0.007 respectively). A statistically significant good correlation was found between these two devices in all parameters except for the LSC in temporal side (*p* = 0.597, r = 0.093). Nevertheless, SS-OCT and SD-OCT had poor agreement in these parameters. The 95% LoA for the nasal/temporal LTM, AOD500, AOD750 and LSC between these two devices were − 154.0 to 257.1/188.3 to 242.5 μm, − 179.8 to 90.5/− 238.4 to 153.3 μm, − 208.7 to 100.1/− 299.7 to 233.2 μm and − 52.5 to 88.2/− 78.7 to 131.0 μm, respectively.

## Discussion

The identification of the SS is crucial in angle assessment, as it offers a reference point of discerning trabecular meshwork and serves as a landmark for quantitative measurements such as AOD500/750 and the LTM [4]. Our study demonstrated impressive identification ability of the SS at nasal and temporal quadrants using SS-OCT compared with SD-OCT. Since the central wavelength of SS-OCT is 1310 nm, it is endowed with a powerful penetrability (6 mm in depth) to detect deeper structures. Satisfactory visualization of the SS using SS-OCT was also achieved by the study of Cumba et al. [8]. Additionally, they reported good interobserver reproducibility of SS-OCT for SS identification, especially in temporal (87%) and nasal (81%) quadrants. Similarly, time-domain OCT with an equal central wavelength also presents an advantage of detecting the SS. In the study by Leung et al. [2] SS can be seen in 98.0% (nasal) and 85.7% (temporal) of the subjects using slit-lamp OCT and in 98.0% (nasal) and 96.0% (temporal) of the subjects using Visante OCT. On the other hand, considering the employment of a superluminescent diode laser wavelength of 840 nm, SD-OCT falls short of clear visibility of deep tissue structures such as the SS. However, it is possible for SD-OCT to identify more superficial structures such as the SL, as we found in our study. Due to this feature, new methods of angle quantification with reference to the SL was proposed by Cheung et al. [9].

**Fig. 5:**
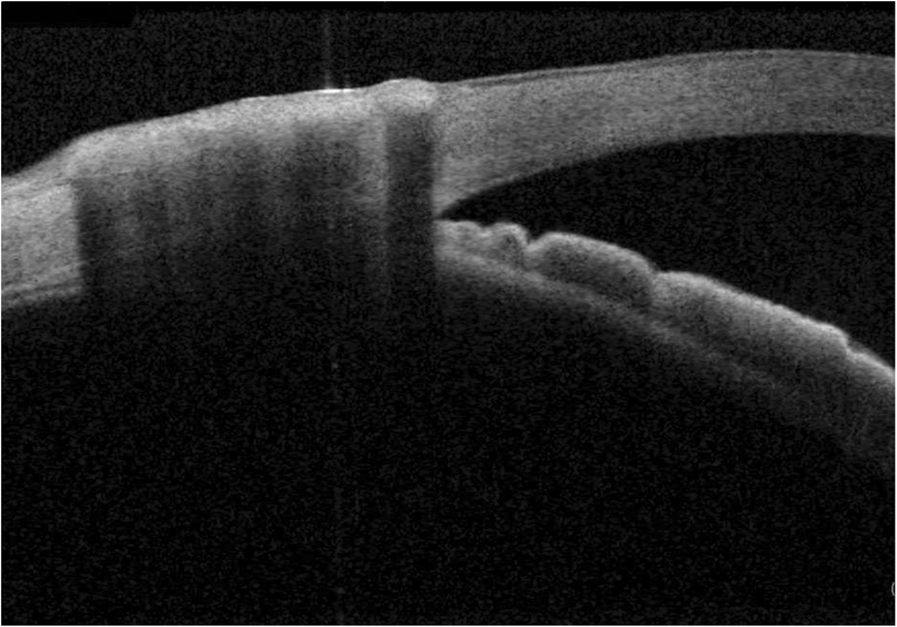
Example of reduced scleral transparency caused by pinguecula

Opinions divide when it comes to the identification of the SC. Usui et al. [10] disagreed with Asrani et al. [11] concerning the morphology and location of the SC. The latter claimed that SC was an arched-shape black space that was located two thirds of the corneal thickness from the corneal surface at the limbus. Conversely, Usui et al. made an argument that analysis of OCT images of the angle structure was easily interfered with the coexistence of the cornea, sclera, SC, and trabecular meshwork which have different reflection and polarization properties [10]. According to their criteria of SC identification, 60.0% (nasal) and 63.3% (temporal) of the SC in subjects’ right eyes were completely observable. The same statistics for the fellow eyes were 90.0% (nasal) and 66.7% (temporal). We basically agreed their definition of the SC’s morphology, whereas, their data were clearly inconsistent with the conclusion we drew. We attributed this disagreement to the different age range of tested subjects. Participants of our study were all above 50 years old whereas the subjects’ age ranging from 29 to 81 in Usui’s study. The transparency of cornea and sclera decreases as we age. For example, the development of pinguecula and pterygium could significantly interfere the visibility of underlying structures. (Fig. 5) In our study, there were no significant differences in discerning capability of the SC between SS-OCT and SD-OCT both at nasal and temporal quadrants (*p* = 0.2810, *p* = 1.0000). Although, with a shorter wavelength, it should be more difficult to view the angle recess. However, Aung T et al. [12] reported good visualization of angle structures including the SC by SD-OCT with certain image processing. Considering the disparities among studies mentioned above, we hold that further studies should be dedicated to standardize the identification and measurement of the SC.

**Fig. 6:**
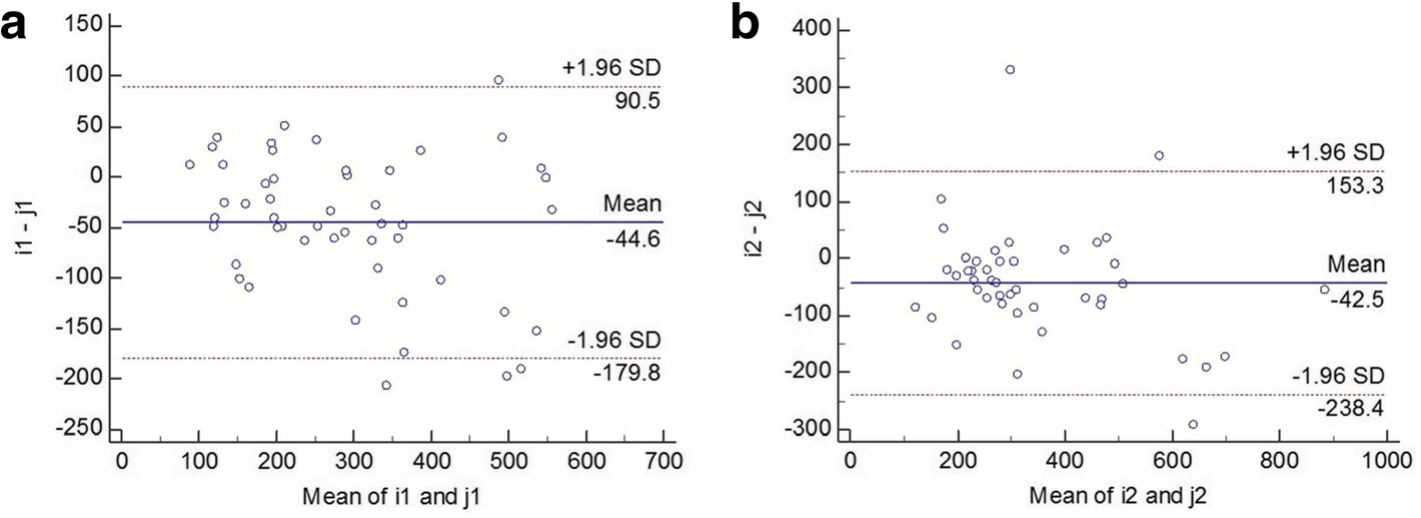
Bland-Altman plots of AOD500 difference between SS-OCT and SD-OCT. **a** demonstrates the nasal quadrant and **b** demonstrates the temporal quadrants. (i1: AOD500 measured by SS-OCT nasally; i2: AOD500 measured by SS-OCT temporally; j1: AOD500 measured by SD-OCT nasally; j2: AOD500 measured by SD-OCT temporally)

**Fig. 7:**
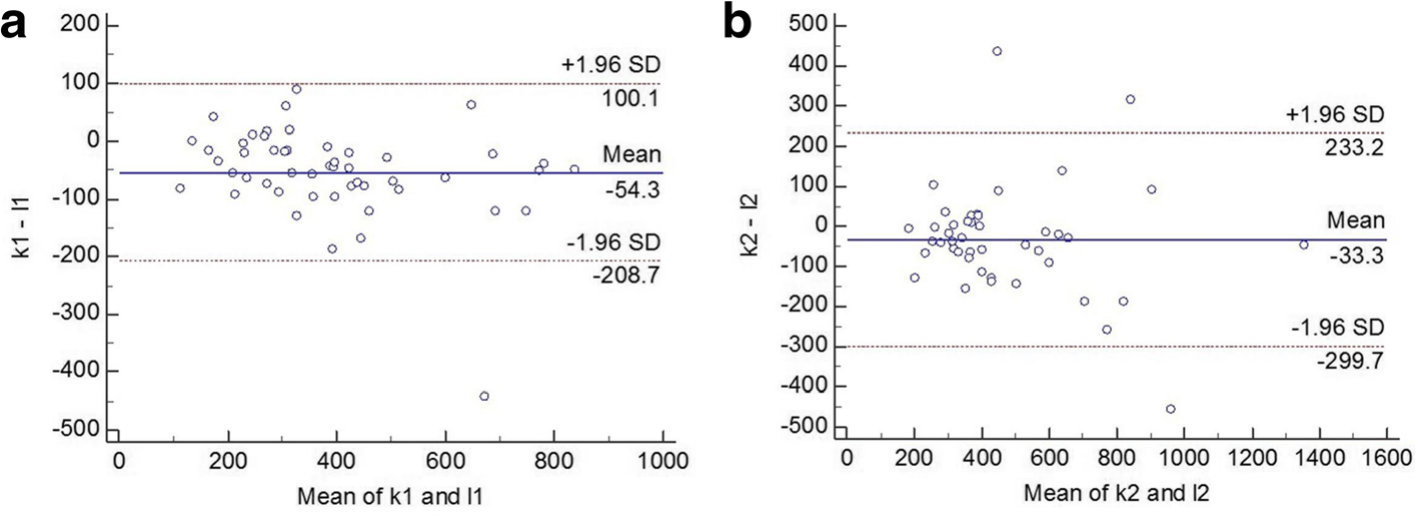
Bland-Altman plots of AOD750 difference between SS-OCT and SD-OCT. **a** demonstrates the nasal quadrant and **b** demonstrates the temporal quadrants. (k1: AOD750 measured by SS-OCT nasally; k2: AOD750 measured by SS-OCT temporally; l1: AOD750 measured by SD-OCT nasally; l2: AOD750 measured by SD-OCT temporally)

Our study demonstrated significant difference in the measurements of AOD500/750 between the two devices. For both OCTs, the temporal data were larger than the nasal ones. And for both quadrants, the measurements were larger using SD-OCT. The former finding could be supported by many other published results. Using slit lamp OCT and Visante OCT, Leung et al. reported that the nasal AOD500 were 534 ± 234 μm / 527 ± 249 μm, while the temporal AOD500 were 628 ± 254 μm /572 ± 275 μm [2]. Similar conclusions were also drawn by Pettersson et al. who measured the ACA in four meridians (0°, 94°, 180°, 274°) with the Sirius Scheimpflug camera and found the mean nasal angle was 40.895 ± 6.908 degrees while the temporal 47.531 ± 5.578 degrees [13]. As to the latter conclusion, previous studies have been concentrated on the agreements between different instruments in angle quantification. Radhakrishnan et al. [14] showed that TD-OCT was similar to UBM in quantitative measurements of the angle such as AOD500 and Trabecular-iris space area (TISA) 500. Pan et al. [15] and Akil et al. [16] demonstrated in their study that SD-OCT was able to give consistent Schwalbe’s line-based angle metrics. However, the studies on SS-OCT were relatively limited. In our study, although good correlation of the results between SS-OCT and SD-OCT was found, the analysis of Bland-Altman plots (Fig. 6 and Fig. 7) revealed that the two devices had poor agreements. The spans of 95% limits of agreement for the nasal/temporal LTM, AOD500, AOD750 and LSC between these two devices were 411.1/54.2 μm, 270.3/391.7 μm, 308.8/532.9 μm and 140.7/209.7 μm, respectively. Considering different types of OCTs were compared in this study, some plausible postulations might serve to explain the differences. Firstly, it should be noted that the refraction of light at the anterior and posterior surface of the cornea leads to the distortion of angle measurements. Both OCT devices adopted a “dewarping” algorithm for the correction of these distortion, so the difference in algorithm (e.g. refractive indexes) should be considered. Secondly, since both devices use external target lights, the difference in illumination might contribute to the phenomenon. Unfortunately, after consulting with the manufacturers’ representatives, we still couldn’t get the exact illumination for the two instruments. Additionally, the distance between the light source and the tested eye could induce disparities in accommodation state, which could affect the lens position and the pupil size.

There were some limitations to this study. Firstly, the participants were all healthy subjects with normal angle conditions. The discerning ability of angle structures under ocular pathologies by different OCT devices was beyond our concern, which confined the extension of the conclusions. Secondly, the subjects were all above 50 years old. It remains to be verified whether the same conclusions can be drawn from younger populations. Thirdly, although the exact brightness of the external fixation lights of two OCT instruments was gauged, it’s still unclear to what extent the measurement of ACA was effected. Since the fixation light directed the eyes of subjects to tilt for a certain angle so as to gain optimal visualization of temporal or nasal anterior chamber, the diameter of pupils remained unmeasurable.

Since their first introduction to the assessment of angle structures, different generations of AS-OCTs are now commercially available. Preceding studies have been conducted focusing on the measurement of the ACA. The current study comprising a relatively large number of consecutive patients and offered a new perspective about the value of SD-OCT and SS-OCT when it comes to angle evaluation.

## Conclusions

In conclusion, we compared the qualitative and quantitative measurements obtained from SD-OCT (RTVue, Optovue Corporation, Fremont, CA) and SS-OCT (SS-1000 CASIA, Tomey, Nagoya, Japan) in Chinese population. SS-1000 CASIA, as the representative to SS-OCT with longer central wavelength demonstrated excellent visualization of the SS, the landmark and reference point for angle measurement. However, the two devices barely distinguished itself from each other as to the identification of the SL and the SC. In the case of the SC, we found that the detection rate was higher at the temporal quadrant compared to the opposite side, regardless of the type of AS-OCT. Further optimization of the SC morphology under OCT scanning might contribute to the standardization of clinical findings. The measurement of angle (AOD500/750) showed significant difference between the two methods and the two quadrants. The poor agreements between SS-OCT and SD-OCT indicated that the data measured from these devices were not interchangeable, although good correlation of the results between the two devices was found. For clinicians and researchers, it is recommended that choices between different OCTs are made based on individual requirement. For example, SS-OCT displayed a better performance in detecting deeper structures. So one might prefer SS-OCT to SD-OCT when examining the scleral spur.

## Data Availability

All data included in this study are available upon reasonable request by contact with the corresponding author.

## Abbreviations

ACA: Anterior chamber angle
AOD: Angle opening distance
AS-OCT: Anterior segment optical coherence tomography
FD-OCT: Fourier-domain optical coherence tomography
LoA: Limits of agreement
LSC: the length of the Schlemnn’s canal
LTM: The length of the trabecular meshwork
SC: The Schlemm’s canal
SD-OCT: Spectrum domain optical coherence tomography
SL: The Schwalbe’s line
SS: The scleral spur
SS-OCT: Swept source optical coherence tomography
TD-OCT: Time-domain optical coherence tomography
UBM: Ultrasound biomicroscopy
